# Hypo and Hyperthyroidism

**Published:** 1915-12

**Authors:** J. S. Hixson

**Affiliations:** San Angelo, Texas


### TEXAS MEDICAL JOURNAL

**Dr. F. E. Daniel, Founder. Established July, 1885**

MRS. F. E. DANIEL, - Publisher and Managing Editor

Published Monthly.—Subscription, $1.00 a Year.

VOL. XXXI AUSTIN, DECEMBER, 1915 No. 7

The publisher is not responsible for views of contributors

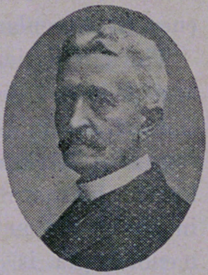

### Original Articles.

For Texas Medical Journal.

### Hypo and Hyperthyroidism.

BY J. S. HIXSON, M. D., SAN ANGELO, TEXAS.

In taking up this subject of hypo and hyperthyroidism, nothing original is set forth in this paper but a short review of some of the facts recently proven by some authors by their experiments.

Lack of development of the thyroid gland retards growth of osseous and muscular systems, and development of a condition of emaciation and chronic cachexia.

The changes in the blood are also remarkable. There is a marked and continued decrease both in the number of red cells and in the amount of hemoglobin, with an increase of leucocytes.

The suppression of the thyroid is followed by a decreased irritability of the sympathetic nervous system, and this is manifested in a sluggishness of the circulation and in certain trophic disturbances. The most contant symptom of thyroid insufficiency in the young growing individual is a remarkable disturbance of the process of growth.

This takes the form of a retardation of growth in height, the growth in breadth continuing very much as in the normal. The arrestation of development produces a dwarf-life appearance, due to imperfect endochondral ossification. A marked arrestation in mental development. This shows itself in decreased locomotion and an impaired intelligence. In adults, where the signs of arrested growth are absent, the most conspicuous symptoms are those of nervous derangement. There are fatigue and pains in the limbs, pronounced lethargy, and, in spite of the fact that the muscular structure is well developed, there is remarkable deficiency in muscular power.

The circulation of the blood is much impaired. The coldness and pallor of the skin are objective signs, as a persistent sensation of chilliness is the subjective sign. The dryness and scaling of the skin, chronic eczema, pigmentating blanching and loss of hair are all signs of trophic disturbances. The changes in the function of the sexual organs are marked. In women, menorrhagia is an unfailing symptom of athyrosis. Markedly cachetic men are sterile. In fact, these patients are old except in years. A special pathogenetic significance is ascribed to thyroid insufficiency in the changes which occur in later life, and which are included in the term senile degeneration. The foundation for the theory that old age results from changes in the thyroid lies in the fact that in old age the thyroid becomes atrophied. This is reinforced by the fact that there is an analogy between the signs of advanced old age and those of myxedema. The falling of the hair, dropping out of the teeth, dry and wrinkled skin, lowered temperature, diminished perspiration, indolent digestion, consequent emaciation, atrophy of sexual organs, decrease of mental power, and the diminution of the activity of the entire nervous system. These are all symptoms which characterize chronic myxedema. Horsley holds the view that senility is due to atrophy and degeneration of the thyroid gland, while myxedema may be described as a condition of premature senility. If this statement be true, we may in our later years partake of thyroid gland and forever remain young. There is no doubt, however, that other ductless glands play as an important rôle in this as the thyroid.

Goiter of adolescence is an edematous condition of the gland, due to watery colloid. This type often disappears with or without treatment. The administration of iodine is very effective in these cases. Operative interference is rarely indicated in these goiters of adolescence unless they enlarge and resist treatment or become encapsulated adenomas. In simple goiter or adenoma of the gland is aggravated by use of iodine. There is no question but that Graves' disease is a chronic malady and only occasionally runs an acute course to termination, and it is only a few months before the heart dilates.

Possibly the most critical period is a few months following the first dilatation of the heart. The diagnosis in this condition is usually easy; however, one should search for further pathology, such as myocarditis affections of the adrenals, pituitary thymus and kidneys.

The secretion of the thyroid is a thin non-stainable substance which contains iodothyrin. The thyroid contains a certain amount of iodine. Less iodine is found in colloid goiter than in the normal. In exophthalmic goiter the iodine is decreased below that of the normal, but in hyperthyroidism iodine is present in excess in the blood. Many cases of hyperthyroidism are transient. Well-marked cases often make a good recovery without medicine, with medicine, or in spite of the various forms of treatment, but the last remedy that was given effected the cure to fail on the next case. We are not always able to estimate the amount of gland to be removed in order to restore favorable conditions. However, the removal of one lobe is usually sufficient, as one-sixth of the thyroid is sufficient to carry on the requirements of the human system, providing it is of good material. The mortality of medicinally treated cases within five years is between 10 per cent and 15 per cent. The mortality from operation has been reduced to 2 per cent to 4 per cent. Besides this, in early operations, we do not have the pathological changes, such as brown atrophy of heart, fatty degeneration of liver, nephritis, insanity, cerebral inco-ordination, etc.

Great harm is nearly always done in treating exophthalmic goiters medicinally, and this is one point that I wish to particularly emphasize in this paper. We may measure the patient's neck each week and assure them that there is no increase in the size of the goiter. We may give them pills, capsules, solutions, applications and X-ray treatments and try to make the patient as well as ourselves believe that we are making progress, but in doing this we have forgotten the pathology that is taking place in other organs, such as brown atrophy of the heart, fatty degeneration of the liver, nephritis, etc., which in a few months or years cripples them for the rest of their lives, or makes them more susceptible to some acute disease which carries them to the great beyond. Therefore, exophthalmic goiter is always a surgical disease.

Adenoma of the thyroid, if producing pressure, causing nervous symptoms, is a surgical case. Even if it is not producing pressure symptoms, at this time it may later cause atrophy of the thyroid, consequently symptoms of hyperthyroidism.

The physiology of the ductless glands, particularly the thyroid, plays an important rôle in our economy, and the time has arrived when we should put more study on the function of these glands.

